# *Effusibacillus dendaii* sp. nov. isolated from farm soil

**DOI:** 10.1007/s00203-021-02470-9

**Published:** 2021-07-07

**Authors:** Tomoyuki Konishi, Tomohiko Tamura, Toru Tobita, Saori Sakai, Namio Matsuda, Hisashi Kawasaki

**Affiliations:** 1 Graduate School of Engineering, Tokyo Denki University, 5, Senju asahi-cho, Adachi-ku, Tokyo, 120-8551 Japan; 2 National Institute of Technology and Evaluation (NITE), 2-5-8, Kazusa-kamatari, Kisarazu, Chiba Japan; 3 Biotechnology Research Center, The University of Tokyo, 1-1-1Bunkyo-ku, Yayoi, Tokyo113-8657 Japan; 4 Collaborative Research Institute for Innovative Microbiology, The University of Tokyo, 1-1-1 Yayoi, Bunkyo-ku, Tokyo, 113-8657 Japan

**Keywords:** *Effusibacillus*, Soil, 16S rRNA, *Alicyclobacillaceae*, Novel species, *Firmicutes*

## Abstract

A Gram-positive, rod-shaped, spore-forming, thermophilic, and acidophilic bacterium, designated as strain skT53^T^, was isolated from farm soil in Tokyo, Japan. Under aerobic conditions, the strain grew at 35–55 °C (optimum temperature 44–55 °C) and pH 4.0–6.0 (optimum pH 5.0). Phylogenetic analysis of the 16S rRNA gene sequence showed that the isolate was moderately related to the type strain of *Effusibacillus consociatus* (94.3% similarity). The G + C content of the genomic DNA was 48.2 mol%, and MK-7 was the predominant respiratory quinone in the strain. The major fatty acids were anteiso-C_15:0_, iso-C_15:0_, and iso-C_16:0_. Based on the phenotypic and chemotaxonomic characteristics, as well as 16S rRNA gene sequence similarity and whole genome analyses, strain skT53^T^ represents a novel species in the genus *Effusibacillus*, for which the name *Effusibacillus dendaii* sp. nov. has been proposed. The type strain is skT53^T^ (= NBRC 114101^ T^ = TBRC 11241^ T^).

**Supplementary Information:**

The online version contains supplementary material available at 10.1007/s00203-021-02470-9.

## Introduction

The family *Alicyclobacillaceae* was established by da Costa and Rainey (2009) to accommodate only the genus *Alicyclobacillus,* which includes spore-forming, acidophilic, and thermophilic bacteria with cellular fatty acids comprising *ω*-alicyclic fatty acids (Wisotzkey et al. 1992). The genera *Kyrpidia* (Klenk et al. 2011) and *Tumebacillus* (Steven et al. 2008) have been added to this family since it was first established. Species belonging to the genus *Kyrpidia*, which contains two species (Bonjour and Arago 1984; Reiner et al. 2018), are spore-forming, thermophilic, and acidophilic; whereas, species belonging to the genus *Tumebacillus* are spore-forming and mesophilic. Although typical bacteria belonging to the genus *Alicyclobacillus* possess *ω*-alicyclic fatty acids, a few members with no detectable *ω*-alicyclic fatty acids have also been identified; among them, two species named *A*. *consociatus* and *A*. *pohliae*, which are phylogenetically distinct from previously known species, have been established (Glaeser et al. 2013; Imperio et al. 2008). Based on 16S rRNA gene sequencing analysis, these two species were subsequently reclassified into the new genus *Effusibacillus* along with *E. lacus* in 2014 (Watanabe et al. 2014). In the present study, a novel strain, termed skT53^T^, isolated from farm soil was taxonomically studied using polyphasic approaches. Herein, we propose a new species of the genus *Effusibacillus*.

## Materials and methods

### Sampling and isolation

Strain skT53^T^ was isolated from the soil of a farm in Adachi-ku Tokyo (35 °47 ′01.3 ″ " N, 139 °47 ′58.0 "″ E), where crop failure occurred in 2014.

Briefly, 1 g of soil sample was suspended in 4.5 mL of sterile saline, and the suspension was allowed to stand for 10 min prior to the recovery of the supernatant. The supernatant was serially diluted and plated on SKT medium containing (per liter) 0.8 mg Difco nutrient broth, 6 g gellan gum, and 0.16 g CaCl_2_. The inoculated plates were incubated at 37 °C for 4 weeks. To select microorganisms that exhibited growth only in oligotrophic media, colonies formed were transferred to both SKT and LB media plates. Microorganisms that showed growth on SKT medium without demonstrating growth on LB medium were selected. Selected microorganisms were suspended in sterile saline and incubated at 80 °C for 30 min to enable the formation of spores, and each suspension was then plated onto SKT medium. After incubation at 37 °C for 4 weeks, growth of nine microorganisms was observed; these were then collected and stored for further analysis. The nine microorganisms were suspended individually in sterile saline, and the cell suspensions were incubated at 80 °C for 30 min to facilitate spore formation. After incubation, sporulation of all nine microorganisms was confirmed using a Wirtz spore staining kit (Muto Pure Chemicals, Japan). The 16S rRNA genes of the nine microorganisms were amplified through PCR with the primers 27f (= 8f) and 1492r (Turner et al. 1999; Loy et al. 2002), and their sequences were analyzed. Among the nine microorganisms, we selected the one with the lowest similarity in 16S rRNA gene sequences compared to those with known sequences and designated it as strain skT53^T^.

### Phenotypic and microscopic analysis and growth conditions

Analysis of cell morphology, assessment using Gram staining, determination of catalase and oxidase activities, and examination of optimal medium conditions were performed by the Identification Service of Techno Suruga Lab Co., Ltd., Japan. Cell morphology was observed using a stereomicroscope (SMZ800N; Nikon, Japan). Gram staining results were observed under a light microscope (BX50F4; Olympus, Japan) using a Gram staining kit (Nissui Pharmaceutical, Japan). Catalase and oxidase activities were determined according to the method described by Barrow and Feltham (1993). To assess anaerobic growth, the growth of the strain skT53 on a yeast extract mineral medium plate (DSM Medium 259, https://www.dsmz.de/collection/catalogue/microorganisms/culture-technology/list-of-media-for-microorganisms) was analyzed using the AnaeroPack System (Mitsubishi Gas Chemical, Japan) (Delaney and Onderdonk 1997).

Each experiment for characterization of strain skT53^T^ was conducted in triplicate at 50 °C (excluding the growth temperature test) using the yeast extract mineral medium with pH adjusted to pH 5.0 (excluding the pH test). To evaluate the optimum growth temperature, cultures were incubated at 11 different temperatures ranging from 30 °C to 80 °C, using liquid media with incubation temperatures ranging from 30 °C to 50 °C as well as solid media with incubation temperatures ranging from 50 °C to 80 °C. To evaluate optimal growth pH, cultures were incubated using solid media with pH adjusted to pH 4.0, 5.0, 6.0, and 7.0, with HCl or NaOH solution according to previously described methods (Sakamoto et al. 2017).

The utilization of organic substrates was assessed using a liquid medium containing (per liter) 1.0 g MgSO_4_.7H_2_O, 0.1 g CaCl_2_.2H_2_O, 0.1 g NH_4_Cl, 0.1 g KH_2_PO_4_, 0.1 g KCl, 1 mL trace element solution, 1 mL selenite tungstate solution, 1 mL vitamin mixture, 1 mL vitamin B_12_ solution, and 1 mL thiamine solution (Watanabe et al. 2014), buffered with 20 mM MES-NaOH (pH 5.0) under oxic conditions. All stock solutions were prepared according to the protocol described by Widdel and Bak (1992). Each substrate was added to the defined medium at a final concentration of 10 mM. The substrates tested included carbohydrates (D-glucose, D-galactose, D-arabinose, sucrose, cellobiose, D-fructose, maltose, mannose, melibiose, D-sorbitol, trehalose, D-xylose, and *N*-acetylglucosamine) and organic acids (acetate, fumarate, D-lactate, L-lactate, and succinate). Additionally, yeast extract was tested at a final concentration of 2 g/L. The sugar oxidation test was performed using an API50CH (BioMérieux, France). The enzyme activity test was performed using APIZYM (BioMérieux, France).

### Chemotaxonomic analysis

Menaquinone extraction was performed according to the methods described by Collins et al. (1977), and analysis was performed using HPLC (Kroppenstedt 1982). Polar lipids were extracted from 100 mg of freeze-dried cells, purified using the methods described by Minnikin et al. (1979), and analyzed via thin-layer chromatography using chloroform/methanol/water (65: 25: 4, by volume) in the first direction and chloroform/acetic acid/methanol/water (80: 18: 12: 5, by volume) in the second. Cellular fatty acid methyl esters were identified and quantified by gas chromatography (6890 N; Agilent Technologies, USA) according to the standard protocol of the Sherlock Microbial Identification System (Sasser 1990) with the Sherlock Midi software (version 6.2) and the TSBA6 database. Amino acids of peptidoglycans were analyzed as described previously (Hamada et al. 2012). The isomer of diaminopimelic acid (DAP) in the cell wall peptidoglycan was determined as described by Hasegawa (1983).

### The 16S rRNA gene sequencing and phylogenetic analysis

The 16S rRNA gene fragment was amplified using the universal primers 9F and 1510R. The nucleotide sequence of the amplified fragment was determined by Fasmac Co., Ltd. (Japan), using the primers 9F, 515F, 1099F, 536R, 926R, and 1510R (Lane et al. 1985; Turner et al. 1999; Lane 1991; Nakagawa et al. 2001). The almost-complete 16S rRNA gene sequence (1471 nt) of strain skT53^T^ was compared with that of the type strains of species with valid published names using EzBioCloud (Yoon et al. 2017a). The CLUSTAL X program (Thompson et al. 1997) was used to align the 16S rRNA gene sequence of strain skT53^T^ with the corresponding sequences of the family *Alicyclobacillaceae*. Phylogenetic trees were reconstructed using the neighbor-joining (NJ) (Saitou et al. 1987), maximum-likelihood (ML) (Felsenstein 1981), and maximum-parsimony (MP) (Fitch 1971) algorithms using the MEGA X program (Kumar et al. 2018). The resultant tree topologies were evaluated by performing bootstrap analysis (Felsenstein 1985) based on 1000 replicates.

### Genome sequencing and analysis

Genomic DNA extraction of the strain skT53^T^ was conducted using cultured cells and the Wizard® Genomic DNA Purification Kit (Promega, USA). Genome sequencing was performed by Macrogen Japan Co., Ltd. (Japan) using the PacBio RSII. The reads of each strain were assembled using the FALCON-integrated version 2.14. The DNA G + C content of strain skT53^T^ was 48.2 mol%. The consensus phylogenetic tree was constructed based on the data of a multi-locus alignment of core genes in the strain skT53^T^ with related species in the NCBI Assembly database using the automated multi-locus species tree (autoMLST) (https://automlst.ziemertlab.com) (Alanjary et al. 2019).

The average nucleotide identity (ANI) and the digital DNA–DNA hybridization (dDDH) values were used to calculate genomic similarities between strain skT53^T^ and the type strains of other *Effusibacillus* species. The ANI based on BLAST was determined using the ANI Calculator (https://www.ezbiocloud.net/tools/ani) (Yoon et al. 2017b; Goris et al. 2007), and the dDDH values were calculated using the Genome-to-Genome Distance Calculator 2.1 (GGDC; http://ggdc.dsmz.de/distcalc2.php) (Auch et al. 2010; Meier-Kolthoff et al. 2013a, b). Formula 2 was applied to dDDH analysis.

## Results and discussion

The cells of strain skT53^T^ were rod shaped, and Gram staining results indicated that the cells were Gram positive. The diameter of the cells grown on yeast extract mineral medium ranged from 0.6 to 0.8 µm, and their length ranged from 2 to 10 µm. Spores were observed at high temperatures. The phenotypic characteristics of the strain skT53^T^ are shown in Table 1.

**Table 1:** Differential properties of skT53^T^ and representatives of other species in the genus *Effusibacillus* and some other species in the family *Alicyclobacillaceae*

Characteristic	1	2	3	4	5	6
Anaerobic growth	w	+	−	+	−	−
Optimal growth temperature	44–55	50–52	30	55	60–65	25–30
pH range for growth	4.0–6.0	7.0–8.5	5.5–10.5	4.5–7.5	2.0–6.0	5.5–9.0
Oxidase/catalase	+ / −	+ / −	Weak/ −	− / −	− /Weak	− / −
DNA G + C content (mol%)	48.2	50.8	47	55.1	60.3	53.1
Major fatty acids	anteiso-C_15:0_, iso-C_15:0_, iso-C_16:0_	iso-C_14: 0_, iso-C_15: 0_, iso-C_16: 0_	iso-C_15: 0_, anteiso-C_15: 0_	iso-C_15: 0_, iso-C_16: 0_, iso-C_17: 0_	ω-cyclohexane-C_17: 0_, ω-cyclohexane-C_19: 0_	iso-C_15: 0_
Aerobic growth on:						
D-Glucose	−	+	+	+	+	+
D-Fructose	−	+	−	+	+	+
Cellobiose	−	+	−	+	+	+
Sucrose	−	−	+	+	+	s
Melibiose	−	−	−	+	+	s
*N*-Acetylglucosamine	−	+	+	+	−	s

Strains: 1, skT53^T^; 2, *Effusibacillus lacus* skLN1^T^; 3, *Effusibacillus consociatus* CCUG 53762^ T^; 4, *Effusibacillus pohliae* CIP 109385^ T^; 5, *Alicyclobacillus acidocaldarius* 104-1A^T^; 6, *Tumebacillus permanentifrigoris* Eur1 9.5^ T^. Data for strains 2–6 were obtained from Watanabe et al. (2014)

+ , growth; − , no growth; w, weak growth; s, slight growth

The skT53^T^ strain did not grow with the sugars tested but grew aerobically on yeast extract, acetate, fumarate, D-lactate and succinate. No sugar was oxidized in the sugar oxidation test using API50CH. The strain showed weak anaerobic growth under the conditions described in the Materials and methods. Evaluation of enzyme activity using APIZYM revealed that the strain skT53^T^ exhibits alkaline phosphatase, esterase (C4), esterase lipase (C8), leucine allyl amidase, acid phosphatase, and naphthol-AS-BI-phosphohydrase activities.

The major cellular fatty acids (> 10% of the total) of strain skT53^T^ were anteiso-C_15:0_ (18.26%), iso-C_15:0_ (17.40%), and iso-C_16:0_ (15.36%); the total cell fatty acid profile of the strain is shown in Table 2. The respiratory quinones in the strain were MK-7 (97.5%) and MK-8 (2.4%). Polar lipids included diphosphatidylglycerol, phosphatidylglycerol, phosphatidylethanolamine, phosphatidylmethylethanolamine, three unidentified phospholipids, and two unidentified polar lipids. The peptidoglycan sample contained alanine, glutamic acid, and *meso*-diaminopimelic acid. All chemotaxonomic data are consistent with the description of the genus *Effusibacillus.*

**Table 2:** Cellular fatty acid contents (percentage of total) of strain skT53^T^ and the closest relatives

Fatty acid	1	2	3	4
iso-C_13:0_	0.52	−	−	−
iso-C_14:0_	7.99	24.93	7.5	3.0
C_14:0_	0.63	1.98	0.8	−
iso-C_15:0_	20.14	14.93	12.9	39.4
anteiso-C_15:0_	25.85	11.3	20.4	10.3
C_15:0_	−	−	−	2.4
C_15:1_ ω6c	0.38	−	−	−
iso-C_16:1_ H	0.38	1.88	8.4	−
iso-C_16:0_	17.95	36.31	−	14.0
C_16:1_ ω5c	−	−	1.5	−
C_16:0_	7.00	5.86	3.9	2.7
iso-C_17:0_	5.31	2.05	7.5	19.6
iso-C_17:0_ ω9c	−	−	0.9	−
anteiso-C_17:0_	2.36	0.76	2.7	6.2
C_17:1_ ω6c	0.56	−	−	−
C_17:0_	8.05	−	−	−
iso-C_18:1_ H	−	−	1.9	−
iso-C_18:0_	0.46	−	−	−
C_18:0_	0.63	−	−	−
C_18:1_ 2OH	0.46	−	−	−
Summed Features*				
Sum In Feature 3	0.42	−	−	−
Sum In Feature 4	0.92	−	−	−

Strains: 1, skT53^T^; 2, *Effusibacillus lacus* skLN1^T^; 3, *Effusibacillus consociatus* CCUG 53762^ T^; 4, *Effusibacillus pohliae* CIP 109385^ T^. Data for strains 2, 3, and 4 were taken from Watanabe et al. (2014) and Glaeser et al. (2013). − , Not detected/not reported

^*^Summed features are groups of two or three fatty acids that cannot be separated by GLC using the MIDI system. Summed feature 3 comprises C_16:1_ ω7c/C_16:1_ ω6c. Summed feature 4 comprises C17:1 iso I / anteiso B

**Fig. 1:**
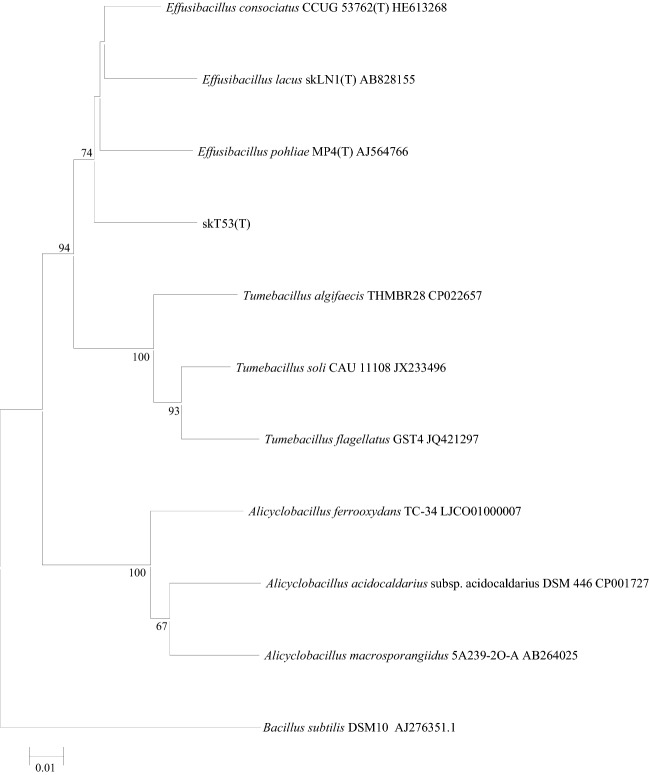
Phylogenetic tree based on 16S rRNA gene sequences created using the neighbor-joining method in MEGA X (Kumar et al. 2018), showing the position of strain skT53^T^ and type strains within the family *Alicyclobacillaceae*. Numbers at branching points refer to percentages of bootstrap values over 50% derived from 1000 replications. Bar, 0.01 substitutions per nucleotide position

**Fig. 2:**
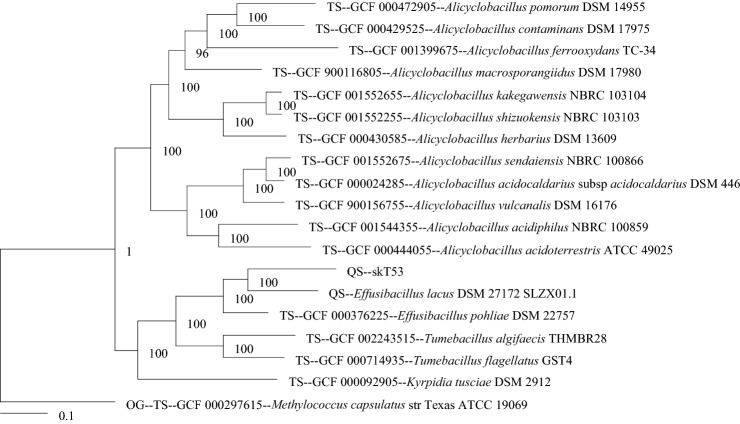
The consensus phylogenetic tree was built from a multi-locus alignment of core genes in strain skT53^T^ with *Alicyclobacillaceae* species in the NCBI Assembly database by using the automated multi-locus species tree (autoMLST) (https://automlst.ziemertlab.com)

The 16S rRNA gene sequence indicated that the species most closely related to strain skT53^T^ were *E. consociatus* CCUG53762^T^ strain (94.3%; Glaeser et al. 2013), *E. lacus* skLN1^T^ (93.4%; Watanabe et al. 2014) and *E. pohliae* MP4 (93.5%; Imperio et al. 2008). Strain skT53^T^ showed less than 91.3% 16S rRNA gene sequence similarity with other members of the family *Alicyclobacillaceae*. These results are also in line with the classification of strain skT53^T^ in the genus *Effusibacillus*. Phylogenetic analysis based on the 16S rRNA gene sequences revealed that strain skT53^T^ forms a clade with members of the genus *Effusibacillus* supported by a high bootstrap value (Fig. 1) and ML and MP algorithms (Supplemental Fig. S1 and S2). As shown in Fig. 1 (NJ), strain skT53^T^ formed a clade with members of the genus *Effusibacillus*, and the genera *Alicyclobacillus*, *Tumebacillus*, and *Effusibacillus* formed independent clades. In Supplemental Fig. S1 (ML) and S2 (MP), strain skT53^T^ formed a clade with members of the genera *Effusibacillus* and *Tumebacillus*. Members of the genus *Tumebacillus* formed a daughter clade against the genus *Effusibacillus*. These results show that strain skT53^T^ phylogenetically belongs to the genus *Effusibacillus*. In addition, these results based on the 16S rRNA gene sequences were consistent with the phylogenetic analysis based on the multi-locus alignment of core genes (Fig. 2).

Strain skT53^T^ exhibited dDDH values of 22.5% for *E. lacus* DSM 27172^ T^ and 18.8% for *E. pohliae* DSM 22757^ T^; both values are clearly below the 70% threshold for the definition of bacterial species (Wayne et al. 1987). Strain skT53^T^ exhibited an ANI of 72.0% for *E. pohliae* DSM 22757^ T^ and 71.8% for *E. lacus* DSM 27172^ T^. These results are significantly below 95–96% (Goris et al. 2007) recommended cut-off points for ANI values.

Based on the data presented, we conclude that strain skT53^T^ represents a novel species of the genus *Effusibacillus*, for which the name *E. dendaii* sp. nov. is proposed.

### Description of *Effusibacillus dendaii* sp. nov.

*Effusibacillus dendaii* (den.dai’i. N.L. gen. masc. n., Dendai’s dendaii (the Japanese abbreviation name of Tokyo Denki University, where this isolate was initially characterized).

The isolate is a facultatively anaerobic, gram-positive, spore-forming, and rod-shaped bacterium with a cell size of 0.6–0.8 × 2–10 µm. The temperature range for growth is 35–55 °C, and the optimum growth temperature is 44–55 °C. The pH range for growth is 4.0–6.0, and the optimum pH was 5.0. It is oxidase positive but catalase negative. It utilizes yeast extract, acetate, fumarate, D-lactate, and succinate as carbon sources. The major fatty acids are anteiso-C_15:0_, iso-C_15:0_, and iso-C_16:0_. The major respiratory quinone is MK-7. The polar lipids comprise diphosphatidylglycerol, phosphatidylglycerol, phosphatidylethanolamine, phosphatidylmethylethanolamine, three unidentified phospholipids, and two unidentified polar lipids. The diagnostic diamino acid in the cell wall peptidoglycan is *meso*-DAP. The in silico genomic DNA G + C content of the type strain is 48.2 mol%.

The type strain, skT53^T^ (NBRC 114101^ T^ = TBRC 11241^ T^), was isolated from farm soil in Adachi-ku, Tokyo, Japan.

## Supplementary Information

Below is the link to the electronic supplementary material.

Supplementary file1 (PPTX 69 KB) Phylogenetic tree based on 16S rRNA gene sequences created using the maximum-likelihood method in MEGA X (Kumar et al., 2018), showing the phylogenetic positions of strain skT53T and type strains within the family Alicyclobacillaceae. Numbers at branching points refer to percentages of bootstrap values over 50% derived from 1000 replications. Bar, 0.02 substitutions per nucleotide position.

Supplementary file2 (PPTX 69 KB) Phylogenetic tree based on 16S rRNA gene sequences created using the maximum-parsimony method in MEGA X (Kumar et al., 2018), showing the phylogenetic positions of strain skT53T and type strains within the family Alicyclobacillaceae. Numbers at branching points refer to percentages of bootstrap values over 50% derived from 1000 replications.
