# Supplementary material for: Effusibacillus dendaii sp. nov. isolated from farm soil
Source: Arch Microbiol. 2021 Jul 7;203(8):4859–65. doi: 10.1007/s00203-021-02470-9 (PMC8502169; doi:10.1007/s00203-021-02470-9)
Supplement: Supplementary file 1 — Supplementary file1 (PPTX 69 KB) Phylogenetic tree based on 16S rRNA gene sequences created using the maximum-likelihood method in MEGA X (Kumar et al., 2018), showing the phylogenetic positions of strain skT53T and type strains within the family Alicyclobacillaceae. Numbers at branching points refer to percentages of bootstrap values over 50% derived from 1000 replications. Bar, 0.02 substitutions per nucleotide position. [file 203_2021_2470_MOESM1_ESM.pptx]

## Slide 1
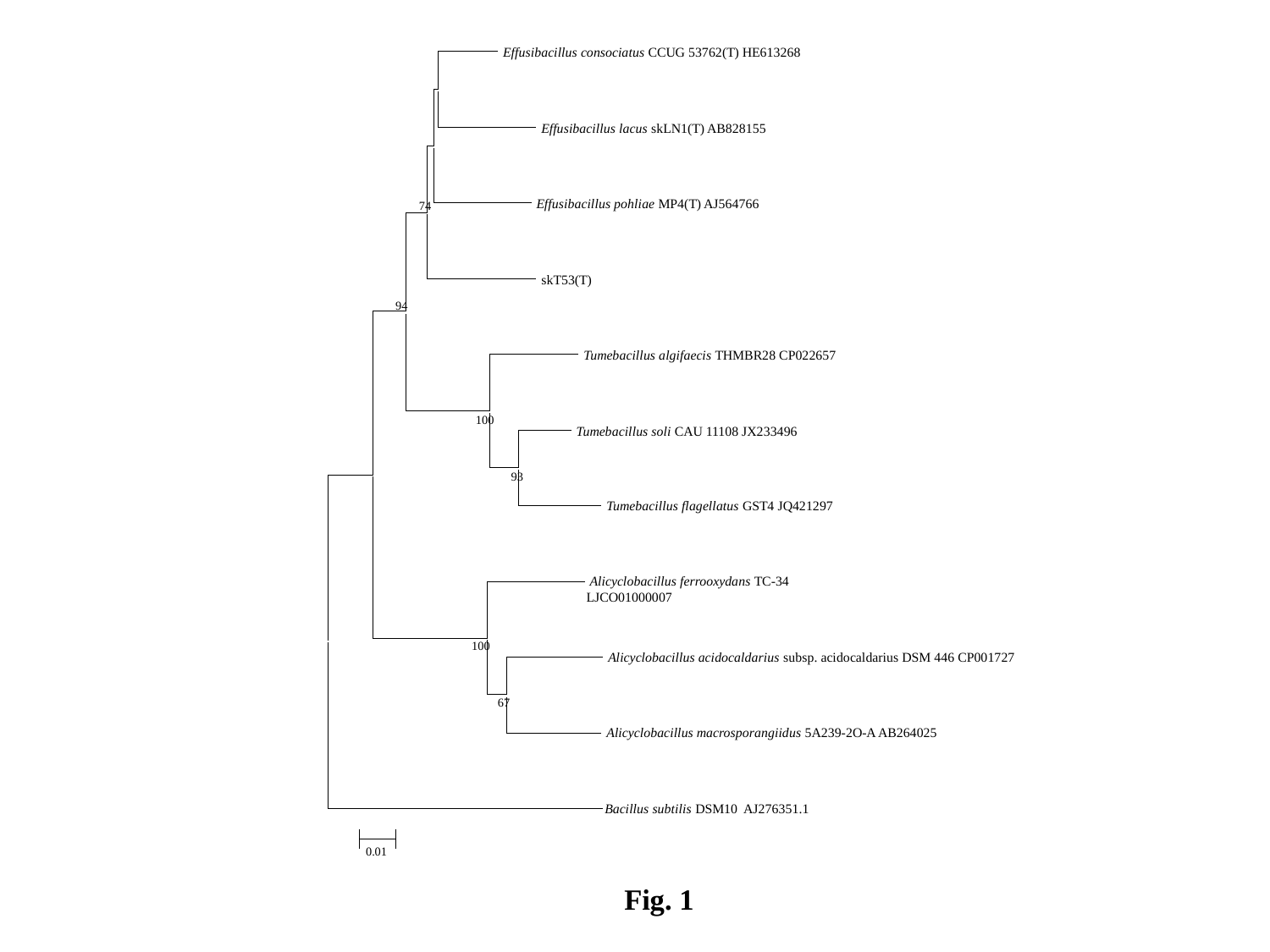

Effusibacillus consociatus CCUG 53762(T) HE613268
 Effusibacillus lacus skLN1(T) AB828155
 Effusibacillus pohliae MP4(T) AJ564766
74
 skT53(T)
94
 Tumebacillus algifaecis THMBR28 CP022657
100
 Tumebacillus soli CAU 11108 JX233496
93
 Tumebacillus flagellatus GST4 JQ421297
 Alicyclobacillus ferrooxydans TC-34 LJCO01000007
100
 Alicyclobacillus acidocaldarius subsp. acidocaldarius DSM 446 CP001727
67
 Alicyclobacillus macrosporangiidus 5A239-2O-A AB264025
Bacillus subtilis DSM10 AJ276351.1
0.01
Fig. 1

## Slide 2
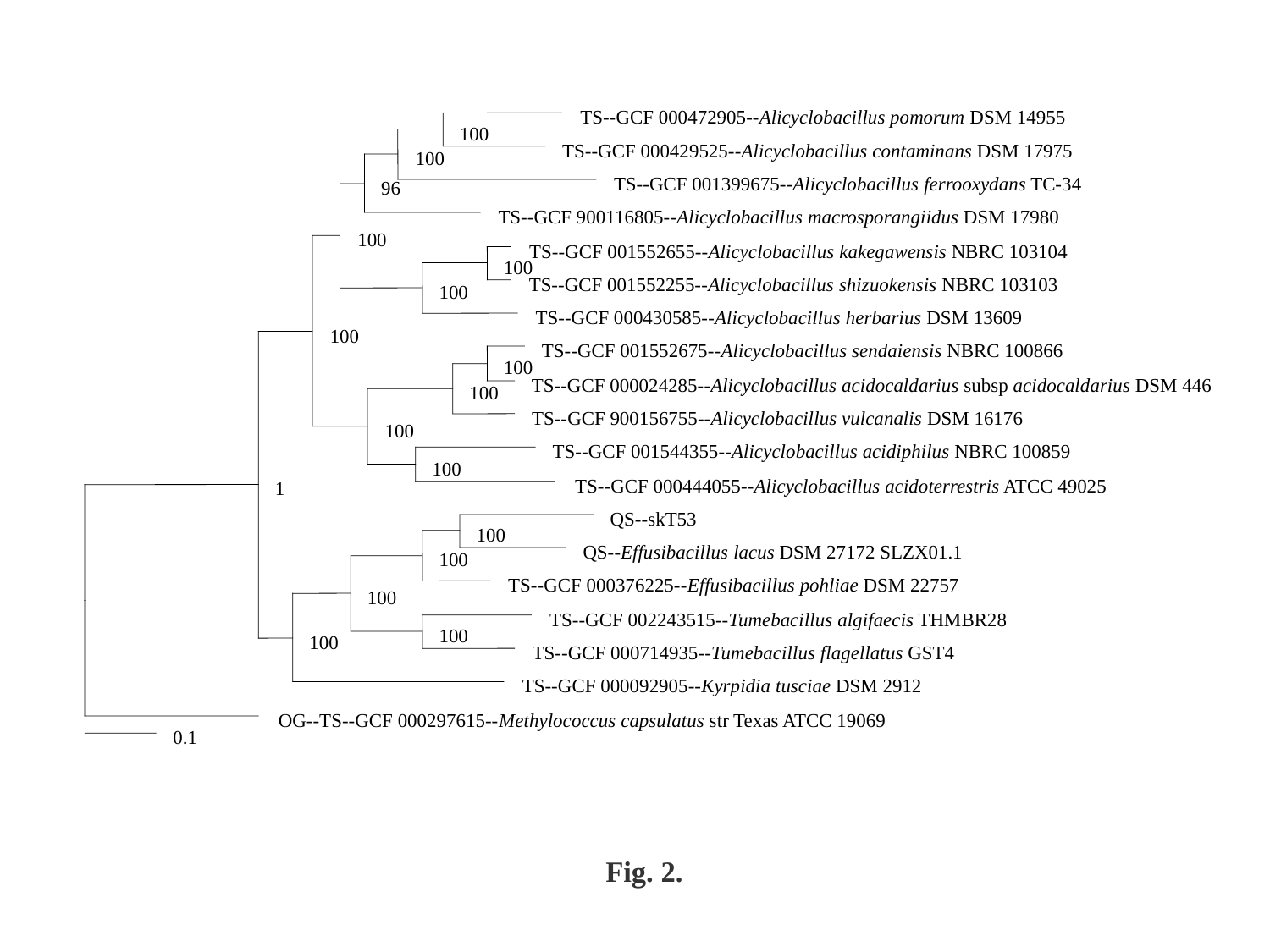

TS--GCF 000472905--Alicyclobacillus pomorum DSM 14955
100
TS--GCF 000429525--Alicyclobacillus contaminans DSM 17975
100
TS--GCF 001399675--Alicyclobacillus ferrooxydans TC-34
96
TS--GCF 900116805--Alicyclobacillus macrosporangiidus DSM 17980
100
TS--GCF 001552655--Alicyclobacillus kakegawensis NBRC 103104
100
TS--GCF 001552255--Alicyclobacillus shizuokensis NBRC 103103
100
TS--GCF 000430585--Alicyclobacillus herbarius DSM 13609
100
TS--GCF 001552675--Alicyclobacillus sendaiensis NBRC 100866
100
TS--GCF 000024285--Alicyclobacillus acidocaldarius subsp acidocaldarius DSM 446
100
TS--GCF 900156755--Alicyclobacillus vulcanalis DSM 16176
100
TS--GCF 001544355--Alicyclobacillus acidiphilus NBRC 100859
100
TS--GCF 000444055--Alicyclobacillus acidoterrestris ATCC 49025
1
QS--skT53
100
QS--Effusibacillus lacus DSM 27172 SLZX01.1
100
TS--GCF 000376225--Effusibacillus pohliae DSM 22757
100
TS--GCF 002243515--Tumebacillus algifaecis THMBR28
100
100
TS--GCF 000714935--Tumebacillus flagellatus GST4
TS--GCF 000092905--Kyrpidia tusciae DSM 2912
OG--TS--GCF 000297615--Methylococcus capsulatus str Texas ATCC 19069
0.1
Fig. 2.

## Slide 3
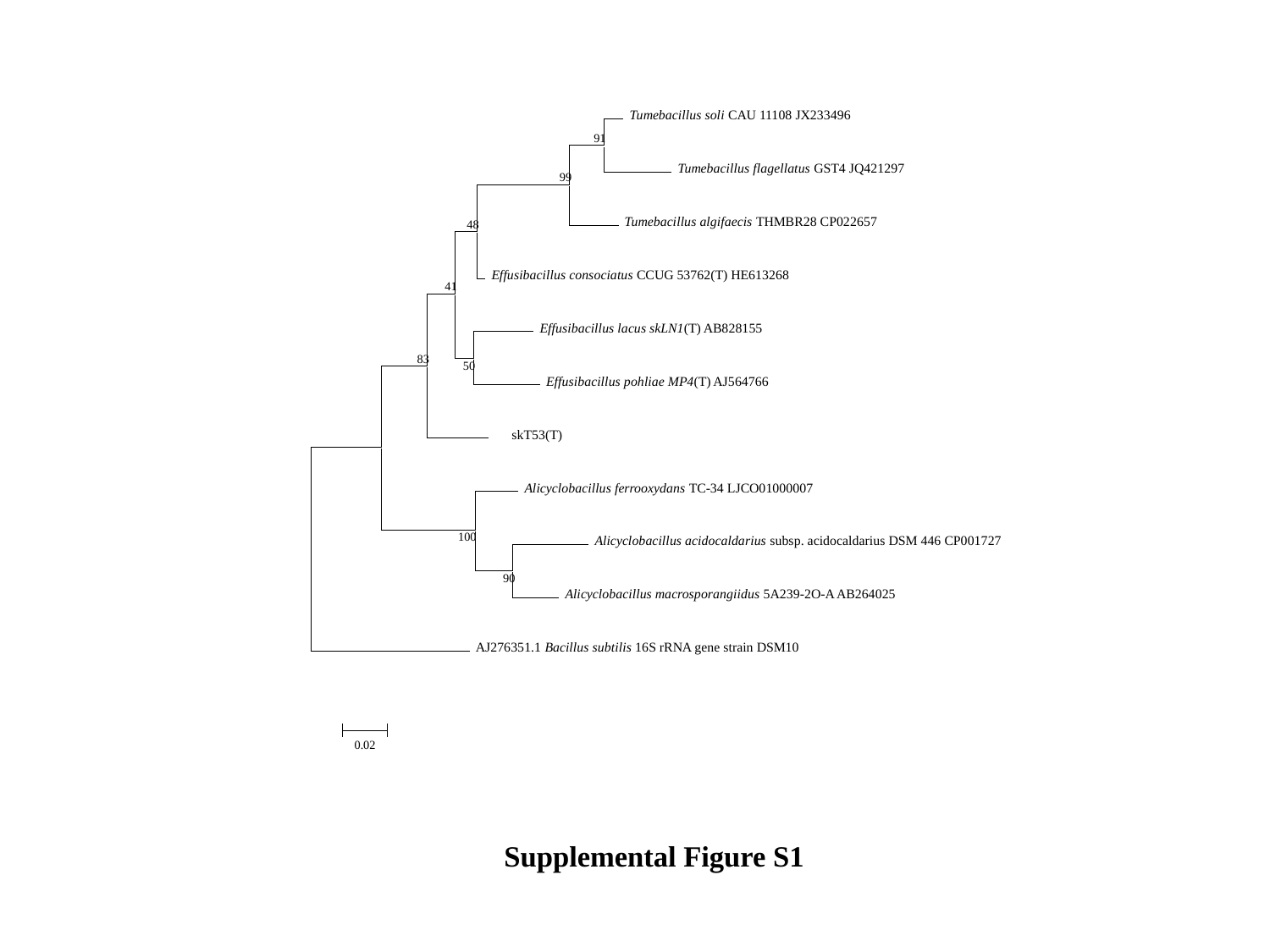

Tumebacillus soli CAU 11108 JX233496
91
 Tumebacillus flagellatus GST4 JQ421297
99
 Tumebacillus algifaecis THMBR28 CP022657
48
 Effusibacillus consociatus CCUG 53762(T) HE613268
41
 Effusibacillus lacus skLN1(T) AB828155
83
50
 Effusibacillus pohliae MP4(T) AJ564766
 skT53(T)
 Alicyclobacillus ferrooxydans TC-34 LJCO01000007
100
 Alicyclobacillus acidocaldarius subsp. acidocaldarius DSM 446 CP001727
90
 Alicyclobacillus macrosporangiidus 5A239-2O-A AB264025
 AJ276351.1 Bacillus subtilis 16S rRNA gene strain DSM10
0.02
Supplemental Figure S1

## Slide 4
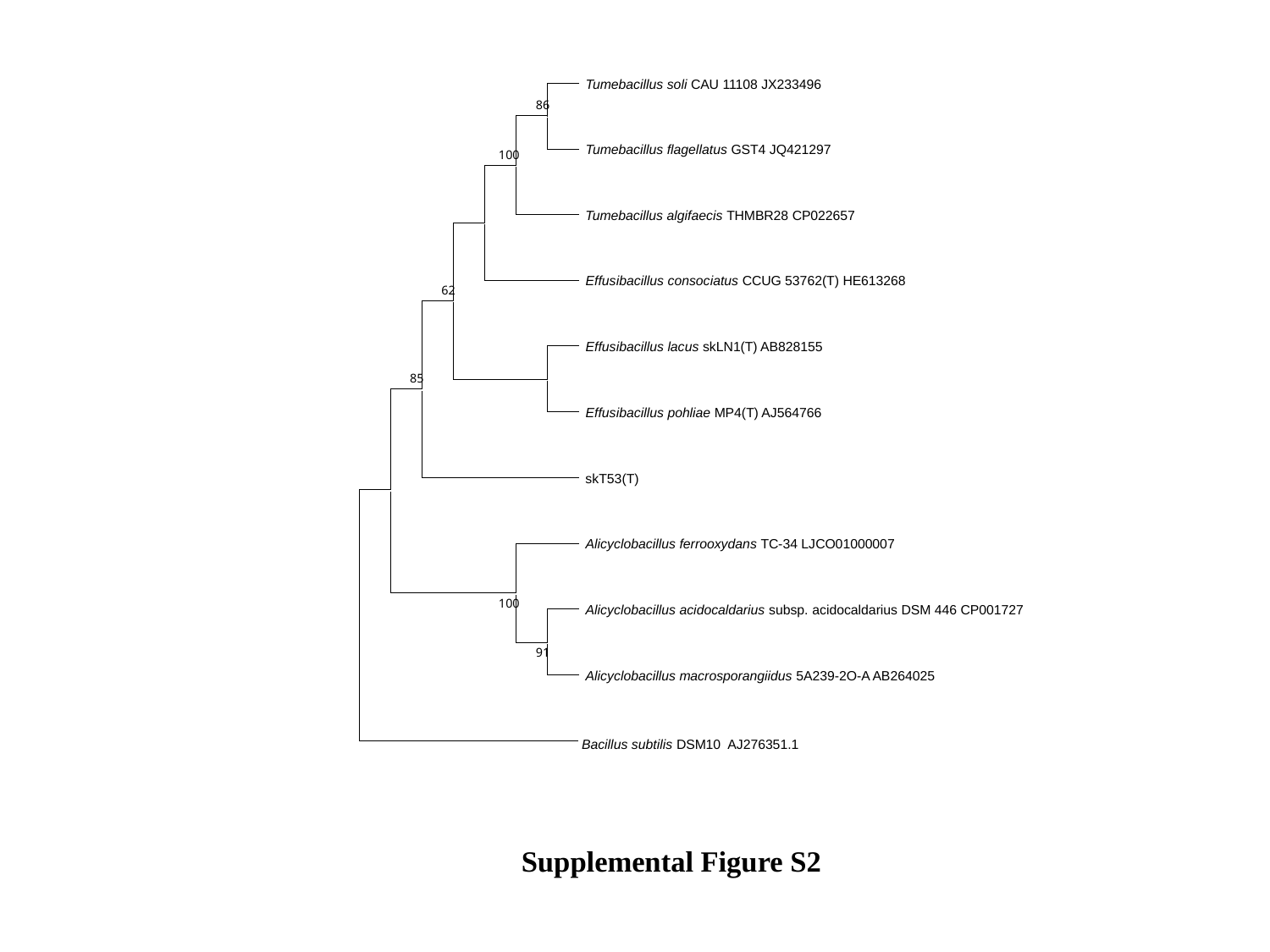

Tumebacillus soli CAU 11108 JX233496
86
 Tumebacillus flagellatus GST4 JQ421297
100
 Tumebacillus algifaecis THMBR28 CP022657
 Effusibacillus consociatus CCUG 53762(T) HE613268
62
 Effusibacillus lacus skLN1(T) AB828155
85
 Effusibacillus pohliae MP4(T) AJ564766
 skT53(T)
 Alicyclobacillus ferrooxydans TC-34 LJCO01000007
100
 Alicyclobacillus acidocaldarius subsp. acidocaldarius DSM 446 CP001727
91
 Alicyclobacillus macrosporangiidus 5A239-2O-A AB264025
Bacillus subtilis DSM10 AJ276351.1
Supplemental Figure S2
